# Impact of body composition parameters on radiation therapy compliance in locally advanced rectal cancer: A retrospective observational analysis

**DOI:** 10.1016/j.ctro.2024.100789

**Published:** 2024-04-27

**Authors:** Giuditta Chiloiro, Marco Cintoni, Marta Palombaro, Angela Romano, Sara Reina, Gabriele Pulcini, Barbara Corvari, Silvia Di Franco, Elisa Meldolesi, Gabriele Egidi, Futura Grassi, Pauline Raoul, Emanuele Rinninella, Antonio Gasbarrini, Maria Cristina Mele, Maria Antonietta Gambacorta

**Affiliations:** a UOC Radioterapia, Dipartimento di Diagnostica per Immagini, Radioterapia Oncologica ed Ematologia, Fondazione Policlinico Universitario A. Gemelli IRCCS, Largo A. Gemelli 8, 00168 Rome, Italy; b UOC di Nutrizione Clinica, Dipartimento di Scienze Mediche e Chirurgiche, Fondazione Policlinico Universitario A. Gemelli IRCCS, Largo A. Gemelli 8, 00168 Rome, Italy; c Centro di Ricerca e Formazione in Nutrizione Umana, Università Cattolica del Sacro Cuore, 00168 Rome, Italy; d Dipartimento di Medicina e Chirurgia Traslazionale, Università Cattolica del Sacro Cuore, 00168 Rome, Italy; e UOC Medicina Interna e Gastroenterologia, Dipartimento di Scienze Mediche e Chirurgiche, Fondazione Policlinico Universitario A. Gemelli IRCCS, Largo A. Gemelli 8, 00168 Rome, Italy; f Dipartimento di Scienze Radiologiche ed Ematologiche, Università Cattolica del Sacro Cuore, 00168 Rome, Italy

**Keywords:** Locally advanced rectal cancer, Sarcopenia, Body composition, Nutritional care, Neoadjuvant chemo-radiotherapy, Compliance to treatment, Survival outcomes, SMI

## Abstract

**Background:**

The impact of body composition and sarcopenia in locally advanced rectal cancer (LARC) is still unclear, even several studies have been published on this issue. Our study aims to analyze the impact of sarcopenia on neoadjuvant chemoradiotherapy (nCRT) tolerance and survival outcomes.

**Methods:**

This is a retrospective, monocentric study where LARC patients treated between 2010 and 2020 were enrolled. A single slice, from the pre-therapy simulation computed tomography (CT) scan, was used to perform the body composition analysis with dedicated software. The primary endpoint was the impact of body composition on radiotherapy (RT) interruption secondarily on overall survival (OS), disease-free survival (DFS), and local control (LC).

**Results:**

The study included 628 LARC patients (40.9 % female, mean age 63.4 years): 24 % had low skeletal muscle index (SMI), 30 % had low muscle density (MD) and 17 (10.3 % of obese) were sarcopenic obese. Higher BMI (OR 2.38, 95 % CI 1.36–4.01) and lower SMI (0.73, 95 % CI 0.55–0.94) resulted as independent predictors of RT interruption. Sarcopenic obesity (HR 2.83, 95 % CI 1.24–6.45) was related to worse OS, while MD (0.96, 95 % CI 0.93–0.98), and higher SMI (0.97, 95 % CI 0.95–0.99) were related to better OS; a lower MD remained also associated even in adjusted multivariable analysis (0.96, 95 % CI0.93–0.98). Moreover, higher visceral adipose tissue (VAT) resulted associated with worse DFS (1.02, 95 % CI 1.01–1.03), while higher SMI was related to better Local Control (0.96, 95 % CI 0.93–0.99).

**Conclusions:**

Body composition analysis, particularly of muscle and fat masses, may be a useful tool for better management of LARC patients undergoing RT. Increased collaboration between radiation oncologists and clinical nutritionists is advisable, to enable early nutritional support of LARC.

## Highlights

• Higher Body Mass Index and lower Skeletal Muscle Index resulted as independent predictors of radiotherapy interruption.
• A higher Muscle Radiodensity was related to better overall survival.
• A higher Visceral Adipose Tissue results associated with worse disease-free survival.
• A higher Skeletal Muscle Index was related to better Local Control.
• Early nutritional support is highly advisable in locally advanced rectal cancer undergoing radiotherapy.

## 1 Introduction

Rectal cancer (RC) is the eighth most common cancer worldwide, with an age-standardized rate of 1.73 per 100.000 person-year [1]. The standard treatment for locally advanced rectal cancer (LARC) involves preoperative chemoradiotherapy (nCRT) followed by total mesorectal excision (TME) [2]. Despite improvements in these therapies, patients with LARC still have a high risk of complications and metastasis, and their response to treatment and prognosis can vary [3]. Therefore, the identification of preoperative risk factors that can be modified could have a significant impact on the risk of complications, prognosis, and tumor response in patients with locally advanced rectal cancer (LARC). This would enable patient stratification for an individualized and integrated care pathway. One of the key factors that can be modified is cancer-related malnutrition. It is a common issue that has been recognized as a crucial predictor of post-operative complications, survival, and tumor response. It results from an unintentional weight loss due to insufficient nutrient intake or absorption. [4]. Malnutrition is present at cancer diagnosis in about 15–40 % of cases and this incidence increases during treatment, characterizing 40–80 % of patients in this phase [5]. Additionally, malnourished patients are more susceptible to chemotherapy-induced toxicities, leading to increased treatment interruptions and reduced treatment efficacy [6]. Definitions of malnutrition, cachexia, and sarcopenia have often not been used consistently in clinical reports, making it difficult to determine their prevalence in cancer patients [7]. Malnutrition is a nosological entity defined as “a state resulting from lack of intake or uptake of nutrition leading to altered body composition (decreased fat-free mass) and body cell mass resulting in decreased physical and mental function and impaired clinical outcome of disease” [8]. In 2016, leading clinical nutrition societies developed the GLIM (Global Leadership Initiative on Malnutrition) Criteria to establish a unified definition for the diagnosis of malnutrition [9]. Malnutrition often involves an imbalance in protein intake and depletion, resulting in a decrease in muscle mass [10]. Sarcopenia, characterized by reduced muscle mass compared to individuals of the same age, gender, and race, is associated with adverse outcomes such as falls, fractures, physical disability, and mortality [11]. In 2018, the European Working Group on Sarcopenia in Older People (EWGSOP2) defined low muscle strength as the main criterion for diagnosing sarcopenia, with muscle strength being the most reliable measure of muscle function [12]. However, the diagnosis of sarcopenia also considers low muscle quantity or quality, and severe sarcopenia is diagnosed when low muscle strength, low muscle quantity/quality, and low physical performance are present [12]. More recently, sarcopenic obesity (SO) has been defined as the coexistence of obesity (BMI greater than 30 kg/m^2^) and sarcopenia by the European Society for Clinical Nutrition and Metabolism (ESPEN) and the European Association for the Study of Obesity (EASO) [13]. Furthermore, several *meta*-analyses have shown the predictive value of specific muscle and fat parameters (body composition) measured by computed tomography (CT) scans for short- and long-term outcomes in various types of cancer [14], [15], [16]. Sarcopenia has been identified as a strong predictor of postoperative complication in LARC patients [17], [18], and in the context of non-metastatic colorectal cancer, depletion of skeletal muscle has been identified as an independent risk factor for survival [19]. Preliminary analysis in the setting of nCRT for rectal cancer suggests a correlation between CT-based muscle mass composition and survival outcomes [20], [21], [22]. The primary aim of this single-center retrospective study is to evaluate the impact of body composition parameters, measured from CT scans obtained during radiotherapy (RT) simulation, in terms of tolerance to nCRT in patients with LARC.

## 2 Materials and methods

Data from LARC patients (cT2-4 and/or cN0-1, cM0) treated between January 2010 and December 2019 at the Fondazione Policlinico Gemelli IRCCS in Rome were retrospectively collected. The study was conducted under the Helsinki Declaration and good clinical practice and was approved by the Ethics Committee of our institution (ID 5022), and informed consent was obtained from patients where appropriate. Inclusion criteria were: (i) histopathologically confirmed diagnosis of rectal adenocarcinoma, (ii) age over 18 years at the time of diagnosis, (iii) simulation CT scan available, (iv) patients undergoing nCRT, (v) surgery performed at least 8 weeks after the end of nCRT, (vi) clinical and pathological staging available, and (vii) follow-up of at least 2 years at the time of analyses. All patients underwent staging by digital rectal examination, colonoscopy, magnetic resonance imaging (MRI) of the pelvic region, and CT scan of the thorax, abdomen, and pelvis with contrast enhancement. Each patient was staged according to the 7th TNM classification [23], and the scheduled treatment was defined by an institutional multidisciplinary tumor board (MTB), consisting of radiation oncologists, surgeons, medical oncologists, radiologists, pathologists, and clinical nutritionists. All patients included in the analysis underwent nCRT with concurrent fluoropyrimidine-based CT with or without weekly oxaliplatin, depending on the stage of the disease [3]. The total dose of RT to the mesorectum in toto and drainage lymphatic nodes, according to disease stage, was 45 Gy/1.8 Gy per day [24]. The dose to the tumor and the corresponding mesorectum was 50.4 Gy with a sequential boost of 5.4 Gy/die or 55 Gy delivered with a concomitant boost of 10 Gy over five weeks, or of 2.2 Gy/die by simultaneous integrated boost (SIB). Radiation treatment was performed with 3-dimensional conformational radiotherapy RT (3D CRT) or intensity-modulated radiation therapy (IMRT) or volumetric modulated arc therapy (VMAT) techniques. Following dosimetric data from treatment plan were collected: Small Bowel V45, Maximal Dose to Bowel (Dmax), PTV1, considered as primary tumour and correspondent mesorectum with a 7-mm isotropic margin, and PTV2 as mesorectum in toto and selected lymphatic drainage stations according to disease stage with a 7-mm isotropic margin. Treatment toxicity was assessed every two weeks during the RT course with clinical evaluation and weekly with blood tests. Toxicity was recorded according to the Common Terminology Criteria for Adverse Events scale (CTCAE) v 5.0 [25]. nCRT treatments were interrupted if the patient showed a toxicity over 3. Clinical response was assessed, at least 6 weeks after the end of nCRT, with pelvic MRI and CT scan. The TME Surgery was performed at least 8 weeks after the end of the nCRT.

In case of major clinic response (mCR) or complete clinical response (cCR) at instrumental re-evaluation with a second MRI scan and endoscopic examination was performed 12–14 weeks after the end of CRT, to confirm the cCR: in these cases, and in selected patients, a conservative approach was opted after MTB evaluation. The pathological response and Tumor Regression Grade (TRG) according to Mandard Criteria [26] was assessed for each patient, and pathological complete response (pCR) was defined as the absence of viable tumor cells in the primary tumor and lymph nodes at the pathological specimen (ypT0 ypN0) TRG 1.

### 2.1 Body composition assessment

**Fig. 1:**
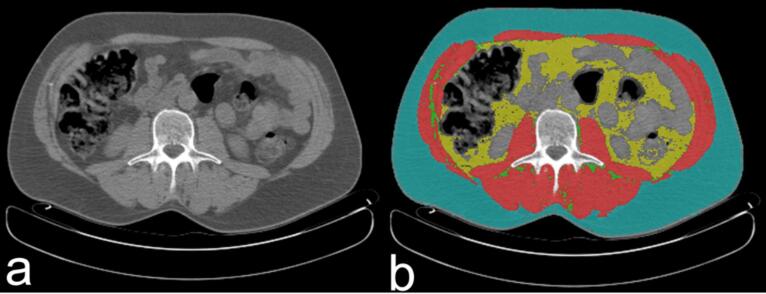
Body Composition Assessment on CT-Scan Slice.

Body composition parameters were analyzed from the CT simulation, by a cross-sectional area analysis at the level of the third lumbar vertebra (L3), using specific dedicated software (SliceOmatic software v5.0- Tomovision, Montreal, Quebec, Canada) which is a medical-specific image analyzing tool, able to recognize different tissue starting from the Hounsfield Units (HU) analysis [16]. The following parameters were assessed: (a) Skeletal Muscle Area (SMA) defined as the total muscle area of bilaterally erector spinae, quadratus lumborum, psoas, internal and external obliques, transversus abdominis, and rectus abdominis; (b) inter-muscular adipose tissue (IMAT) as the adipose tissue within muscular fibers; (c) visceral adipose tissue (VAT) as the adipose tissue presents between bowels and other internal organs; (d) subcutaneous adipose tissue (SAT) as the adipose tissue between the skin and muscular fascia; (e) muscle density (MD) defined as the mean Hounsfield Unit (HU) of SMA (Fig. 1).

Body composition assessment: basal CT-Scan (a); analyzed CT-Scan (b) with muscle mass (red), subcutaneous adipose tissue (blue), visceral adipose tissue (yellow), and intermuscular adipose tissue (IMAT) (green).

Pre-established HU thresholds were used to identify and quantify in cm^2^ each parameter: −29 to + 150 HU for SMA, −190 to − 30 HU for SAT, −150 to − 50 HU for VAT, and − 190 to − 30 HU for IMAT. From those raw data, the skeletal muscle index (SMI), the visceral adipose index (VAI), the subcutaneous (SAI), and the intramuscular adipose index (IMAI) were calculated by normalizing SMA, VAT, SAT, and IMAT for squared height (in m^2^), respectively. According to the sex-specific definitions of Fearon et al., low muscle mass was defined as SMI < 52.4 cm^2^/m^2^ in men and SMI < 38.5 cm^2^/m^2^ in women [27]. For VAT analysis, the low VAT group was defined as VAT < 160 cm^2^ for men and VAT < 80 cm^2^ for women [28], [29].

### 2.2 Statistical analyses

Statistical analyses were performed using STATA® Software (Version 14.0, Stata Corporation; College Station, TX, USA). The normality of the variables was verified through the Kolmogorov–Smirnov test. Quantitative variables are presented through mean ± standard deviation if normally distributed or with the median (interquartile range) in the other cases. The qualitative variables were presented through tables of absolute numbers and percentage frequencies. The primary objective of the study was to evaluate the impact of malnutrition on treatment tolerance in terms of RT interruption. Secondary endpoints were to evaluate the impact of malnutrition: (i) on acute intestinal toxicity (≥G3), (ii) other acute and late complications, (iii) early termination of radiation treatment, (iv) overall survival (OS); (v) disease-free survival (DFS), (vi) metastases free survival (MFS), local control (LC) and (vii) complete response rate after nCRT. Differences among continuous variables were assessed using Student’s *t*-test if variables were normally distributed or the Mann-Whitney test when not normally distributed. Proportions were compared with the Chi-Square test or Fisher exact test, when appropriate. All variables with a p < 0.05 at univariate analysis were considered to perform multivariable logistic analysis to investigate the RT interruption. Survival outcomes were investigated using the Kaplan-Meier method. A multivariable Cox regression model will be constructed, including all variables with p < 0.05 at univariate analysis, to assess differences among these outcomes. Statistical significance was set when p < 0.05 (two-sided tests).

## 3 Results

### 3.1 Baseline Characteristic

Principal demographical and oncological data are shown in Table 1.

**Table 1:** Baseline characteristics and treatment details of study population (628 patients).

Characteristic	Number (%) or Mean ± Standard Deviation
Sex	
Male	371 (59.1)
Female	257 (40.9)
Age	63.4 (±11.4)
cT-stage	
2	56 (9.0)
3	372 (59.1)
4	200 (31.9)
cN-stage	
0	75 (11.9)
N 1–2	553 (88.1)
Extra-mesorectal nodes	167 (26.9)
MRF +	245 (39.4)
Median RT dose (InterQuartile Range)	55 (19.8–58.5)
Small Bowel V45 (cm^3^)	61.0 ± 65.1
Small Bowel Dmax (Gy)	51.5 ± 8.8
PTV 1 vol (cm^3^)	414.0 ± 179.3
PTV 2 vol (cm^3^)	947.8 ± 331.1
Concomitant CT	
5FU/cape	310 (49.4)
5FU/Cape + oxa	293 (46.7)
None	25 (3.9)
RT interruption	152 (23)
Median Interruption (days ± range)	8.8 ± 6.9
Interruption ≥ 5 days	97 (15.4)
CT interruption	228 (36.3)
Acute GI Toxicity	
G1	227 (36.1)
G2	187 (29.8)
G3	110 (17.5)
G4	16 (2.6)
ypT stage	
Tis	12 (1.9)
0	156 (24.8)
1	30 (4.8)
2	142 (22.6)
3	199 (31.7)
4	20 (3.2)
Missing	69 (10.9)
ypN stage	
0	394 (62.7)
1	94 (15.0)
2	21 (3.3)
Missing	119 (18.9)
cCR	107 (17.0)
pCR	154 (24.5)
TRG	
1–2	275 (43.8)
3-4-5	244 (38.9)
Missing	109 (17.3)
Neural invasion	11 (1.8)
Missing	120 (19.1)
Adjuvant Chemotherapy	255 (40.6)
Complete adj CT	208 (81.6)
Watch and wait approach	60 (9.5)
Surgical Resection	
R0	522 (83.2)
R1/R2	31 (4.9)
Missing	75 (11.9)
Late GI Toxicity	252 (40.1)
G1	169 (26.9)
G2	49 (7.8)
G3	31 (4.9)
G4	3 (0.5)

*Abbreviations*: MRF: mesorectal fascia involvement; RT: radiotherapy; GI: gastrointestinal; pCR: pathological complete response; TRG: tumor regression grade; CT: chemotherapy; 5FU/cape: 5Fluorouracil/capecitabine; oxa: oxaliplatin; AR: anterior resection LAR: low anterior resection, APR: abdominal perineal resection; LE/TEM: local excision/ Transanal Endoscopic Microsurgery.

A total of 628 patients met the inclusion criteria and were enrolled and data retrieved for this study. Overall, 372 patients (59.1 %) were a cT3 stage, while 200 (31.9 %) had a cT4 rectal cancer; 553 (88.1 %) patients had a cN 1 or 2 stages, with the26.9 % (1 6 7) patients had extra-mesorectal positive nodes. The median RT dose on GTV was 55 Gy, and 46.7 % of patients received intensified chemotherapy with oxaliplatin and fluorouracil. Overall, 568 (91.6 %) underwent surgery: 136 underwent abdominal-perineal resection, 380 low anterior resection, and 52 local excisions. cCR was reached in 107 patients (17 %), while pCR was achieved in 154 patients (24.8 %); the TRG was collected for 519 patients of whom 275 (52.9 %) showed a good grade of regression (TRG 1 and 2). Surgical resections were classified as: R0, all disease removed with histological confirmation; R1, all gross tumor removed with histologically positive margins; R2, unresected residual disease.Overall, 255 (40.6 %) patients received adjuvant CT.

### 3.2 Body composition analysis

Body composition data are shown in Table 2.

**Table 2:** Body Composition analysis Data of study population.

Body composition characteristics	Number (%)
	628 patients
Weight (kg) ± SD	71.9 ± 14.3
Height (cm) ± SD	165.0 ± 9.6
BMI (kg/m^2^) ± SD	26.3 ± 4.1
Underweight patients (BMI ≤ 18.5 kg/m^2^)	9 (1.4)
Normal weight patients	243 (38.7)
Overweight patients	211 (33.6)
Obese patients (BMI ≥ 30 kg/m2)	165 (26.3)
SMA (cm2) ± SD	133.1 ± 33.3
SMI (cm2/ m2) ± SD	49.9 ± 9.5
Low SMI*	151 (24.0)
MD (HU) ± SD	30.0 ± 8.1
Low MD#	245 (30.0)
IMAT (cm2) ± SD	1074 ± 2581
VAT (cm2) ± SD	174.4 ± 273.2
Low VAT °	251 (40.0)
SAT (cm2) ± SD	198.1 ± 480.1
Sarcopenic Obese patients	17 (2.7 of total; 10.3 of obese patients)

*Abbreviations*: SD: standard deviation; BMI: Body mass index; SMA: Skeletal muscle area; SMI: Skeletal muscle index; MD: Muscle Density; HU: Hounsfield Unit; IMAT: Intramuscular adipose tissue; VAT: visceral adipose tissue; SAT: subcutaneous adipose tissue.

*: SMI < 52.4 cm^2^/m^2^ in men and SMI < 38.5 cm^2^/m^2^ in women.

*^#^: MD < 28.6 HU*.

*°: VAT < 160 cm2 for men and VAT < 80 cm2 for women*.

Mean BMI was 26.3 ± 4.1 kg/m^2^, with a presence of 9 (1.4 %) underweight patients (BMI under 18.5 kg/m^2^), and 165 (26.3 %) obese patients (BMI over 30 kg/m^2^). Among obese patients, 17 (10.3 % of obese, 2.7 % of the total sample) were sarcopenic obese. A correlation between BMI and SMI was found (Pearson coefficient 0.459, p < 0.0001) (Figure S1).

A ROC Curve analysis, applying the Youden method, was constructed to identify an optimal MD cut-off for OS and it was identified at 28.6 HU; 30.0 % of patients had MD under this cut-off. The mean SAT was 198.1 cm^2^, the mean VAT was 174.4 cm^2^, and 251 patients (40.0 %) had a VAT under the pre-defined cut-offs.

### 3.3 Treatment adverse events

Overall, 540 (85.9 %) patients experienced any degree of gastrointestinal (GI) toxicity. In 414 (65.9 %) cases the level of GI disorder was mild or moderate (G1-2), in 110 (15,9%) severe (G3), and in 16 (2.6 %) was life-threatening (G4). RT treatment needed to be interrupted in 152 (23 %) patients with a mean of 8.8 ± 6.9 days and a total of 97 (15.4 %) patients had an interruption of more than 5 days. 229 (36.3 %) patients interrupted concomitant CT for hematological and GI toxicities. Statistical results assessed the association between interruption of RT and anthropometric data, body composition, age, and sex (Table 3), showing that BMI, SMA, SMI, female gender, Small Bowel V45 Volume, and PTV boost volume were correlated with RT discontinuation at univariate analysis.

**Table 3:** RT Interruption.

	Univariate Analysis				Multivariate Analysis	
	No RT Interruption (485 patients)	RT Interruption (137 patients)	p	OR (95 % CI)	OR (95 % CI)	p
Weight (kg)	73.1 ± 14.8	67.7 ± 11.5	***<0.0001***	***0.97*** ***(0.95***–***0.98)***	*Not included*	
Height (cm)	165.7 ± 9.7	162.6 ± 8.8	***0.001***	***0.96*** ***(0.94***–***0.98)***	*Not included*	
BMI (kg/m2)	26.5 ± 4.1	25.6 ± 4.0	***0.027***	***0.94*** ***(0.90***–***0.99)***	***2.38*** ***(1.36***–***4.01)***	***0.001***
Obesity	130 (26.8)	30 (21.9)	0.24			
SMA	137.0 ± 33.2	119.3 ± 30.3	***<0.0001***	***0.98*** ***(0.96***–***0.98)***	1.08 (0.97–1.18)	0.052
SMI	49.5 ± 9.5	45.0 ± 9.2	***<0.0001***	***0.94*** ***(0.92***–***0.96)***	***0.73*** ***(0.55***–***0.94)***	***0.019***
Low SMI*	117 (24.1)	34 (24.8)	0.87			
MD	30.2 ± 7.9	29.1 ± 8.5	0.19			
Low MD#	186 (38.3)	56 (40.9)	0.59			
IMAT	1058 ± 2556	1132 ± 2693	0.76			
VAT	182.9 ± 302.6	147.1 ± 125.7	0.17			
Low VAT°	80 (58.4)	57 (41.6)	0.61			
SAT	190.5 ± 415.1	227.6 ± 669.3	0.42			
Sarcopenic Obesity	13 (2.7)	4 (2.9)	0.77			
Female Sex	181 (37.3)	74 (54.0)	***<0.0001***	***1.97*** ***(1.34***–***2.89)***	0.74 (0.36–1.53)	0.38
Age	63.3 ± 11.6	64.1 ± 9.8	0.65			
MRF	185 (38.1)	60 (43.8)	0.23			
Small Bowel V45 (cm^3^)	58.3 ± 2.7	71.7 ± 7.3	**0.03**	**1.01** **(1.00**–**1.02)**	1.00 (0.98–1.02)	0.11
Small Bowel Dmax (Gy)	51.4 ± 8.7	51.8 ± 0.9	0.61			
PTV 1 vol (cm^3^)	404.5 ± 173.7	446.1 ± 197.1	**0.01**	**1.01** **(1.00**–**1.01)**	1.01 (0.97–1.05)	0.16
PTV 2 vol (cm^3^)	939.2 ± 321.5	986.0 ± 363.8	0.15			

*Abbreviations*: SD: standard deviation; BMI: Body mass index; SMA: Skeletal muscle area; SMI: Skeletal muscle index; MD: Muscle Density; HU: Hounsfield Unit; IMAT: Intramuscular adipose tissue; VAT: visceral adipose tissue; SAT: subcutaneous adipose tissue.

*: SMI < 52.4 cm*^2^*/m^2^ in men and SMI < 38.5 cm^2^/m^2^ in women.

*^#^: MD < 28.6 HU*.

*°: VAT < 160 cm2 for men and VAT < 80 cm2 for women*.

The multivariable analyses confirmed the association for BMI (OR 2.38, 95 % CI 1.36–4.01; *p:0.001*) and SMI (OR 0.73, 95 % CI 0.55–0.94; *p: 0.019*) as independent factors of worst compliance with RT treatment.

### 3.4 Survival outcomes

At a median follow-up of 68 months (95 % CI 42.4–97.4), the OS was 81.2 % (95 % CI 77.4 %-84.4 %) (Figure S2), DFS was 71.7 % (95 % CI 67.4 %-75.5 %) (Figure S3), and LC was 89.9 % (95 % CI 86.8 %-92.3 %) (Figure S4).

The OS appeared to be related to the initial stage of the disease (MRF) and the response to treatment (TRG > 3), but also with the presence of sarcopenic obesity (HR 2.83, 95 % CI 1.24–6.45; *p < 0.01*) (Figure S5), MD (HR 0.96, 95 % CI 0.93–0.98; *p < 0.0001*) (Figure S6), and SMI (HR 0.97, 95 % CI 0.95–0.99; *p < 0.03*).

Interestingly, OS was lower in sarcopenic obese and underweight patients, rather than sarcopenic normal and overweight (Figure S7).

Using multivariable Cox regression model, the following variable were identified as independent prognostic factors: age (HR 1.04, 95 % CI 1.01–1.07; *p < 0.001*), MD (HR 0.96, 95 % CI 0.93–0.98; *p < 0.023*), MRF involvement (HR 1.91 95 % CI 1.24–2.95; *p < 0.003*), and TRG 5 (HR 4.96, 95 % CI 1.12–21.85; *p < 0.001*) (Table 4).

**Table 4:** Overall Survival.

	HR	p	HR	p
Age	***1.05 (1.03***–***1.07)***	***<0.0001***	***1.04 (1.01***–***1.07)***	***0.001***
Female	0.90 (0.62–1.34)	0.61		
Weight	0.99 (0.98–1.01)	0.45		
Height	0.99 (0.97–1.01)	0.84		
BMI	0.98 (0.94–1.03)	0.53		
Obesity	1.13 (0.75–1.69)	0.54		
SMA	0.99 (0.98–1.00)	0.08		
SMI	***0.97 (0.95***–***0.99)***	***0.03***	0.99 (0.95–1.02)	0.43
Low SMI*	0.93 (0.62–1.39)	0.74		
MD	***0.96 (0.93***–***0.98)***	***<0.0001***	***0.96 (0.93***–***0.98)***	***0.023***
Low MD#	***0.63 (0.44***–***0.91)***	***0.001***		
IMAT	0.99 (0.91–1.01)	0.15		
VAT	0.99 (0.99–1.00)	0.41		
Low VAT°	1.21 (0.84–1.73)	0.29		
SAT	0.99 (0.98–1.01)	0.67		
Sarcopenic Obesity	***2.83 (1.24***–***6.45)***	***0.01***	2.24 (0.69–7.21)	0.17
cT				
T2	Ref			
T3	1.10 (0.52–2.30)	0.79		
T4	1.65 (0.78–3.48)	0.19		
Nodes	1.26 (0.68–2.35)	0.45		
Extramesorectal Nodes	1.03 (0.69–1.54)	0.87		
MRF	***1.65 (1.16***–***2.36)***	***0.005***	***1.91 (1.24***–***2.95)***	***0.003***
pCR	0.61 (0.37–1.02)	0.06		
TRG ≥ 3	***1.57 (1.04***–***2.38)***	***0.03***	1.39 (0.91–2.12)	0.13
RT interruption	1.45 (0.97–2.16)	0.07		
Surgical Resection (R1/2 versus R0)	0.76 (0.18–3.10)	0.71		

*Abbreviations*:SD: standard deviation; BMI: Body mass index; SMA: Skeletal muscle area; SMI: Skeletal muscle index; MD: Muscle Density; MRF: MesoRectal Fascia Invasion; HU: Hounsfield Unit; IMAT: Intramuscular adipose tissue; VAT: visceral adipose tissue; pCR: Pathological Complete Response; SAT: subcutaneous adipose tissue; TRG: Tumor Regression Grade.

*: SMI < 52.4 cm*^2^*/m^2^ in men and SMI < 38.5 cm^2^/m^2^ in women.

*^#^: MD < 28.6 HU*.

*°: VAT < 160 cm2 for men and VAT < 80 cm2 for women*.

The DFS was influenced by age, MRF, and features of treatment response as pCR and TRG both at univariate and multivariable analysis. Notably, also VAT was associated with DFS as an independent factor (OR 1.02, 95 % CI 1.01–1.03; *p 0.021*). Interestingly, a low VAT was identified as a predictor of worse DFS only in men (HR 1.65, 95 %CI 1.06–2.61; *p: 0.029*) (Table S1) (Figure S8). LC resulted related with Age (HR 1.03, 95 % CI 1.01–1.06; *p 0.03*), SMI (HR 0.96, 95 % CI 0.93–0.99; *p 0.04*) (Figure S9), and pCR (HR 0.38, 95 % CI 0.16–0.91; *p 0.03*) at multivariable analysis (Table S2).

## 4 Discussion

This study was conducted to evaluate the impact of body parameters assessed with the simulation CT scan to evaluate the impact of body composition parameters on RT interruption and survival outcomes in LARC patients undergoing nCRT. Overall, 24 % of LARC patients had a low muscle mass, in line with the literature [30], [31]. Moreover, we noted that low-muscle mass patients had worse OS while, beyond age and sex, higher SMI showed a protective effect on RT interruption events (OR 0.73). Similar results were obtained in previous publications, highlighting the potential predictive role of body composition assessment and SMI in LARC patients undergoing RT [31], [32]. One of the possible explications of this finding could be the relationship between lower muscle mass and the elevated inflammatory index, detrimental to clinical and survival outcomes [33], [34]. In the CAO/ARO/AIO-04 clinical trial, performed on 1236 rectal cancer-affected patients, Fokas et al. showed that underweight patients had worse DFS, was associated with worse TME quality and higher incidence of acute organ toxicity, and showed higher T stage and reduced ECOG performance status, giving the plausible explanation that underweight is linked to inflammation, a hallmark of cancer with muscle wasting and fat depletion induced by increased levels of cytokines [35]. Starting from these results, a plan of action could be represented by the routinary analysis of body composition using the CT scan images. A correlation between low MD and postoperative complications in LARC patients was observed by Liu and colleagues, finding a correlation between MD and short-term and long-term ileus [36]. Also, *Pekarova et al.* identified a correlation between low psoas density and worse postoperative complications [37]. In our study the survival analyses were performed taking into consideration features related to the patients, tumor, treatment, and body composition: at the multivariate analysis muscular quality, in terms of MD, confirmed its independent prognostic role for OS, even adjusted for age, TRG, and mesorectal fascia involvement (MRF). A very recent study identified low SMI as a strong predictor of worse OS (adjusted HR 3.17; p < 0.001) [28]. Moreover, a *meta*-analysis included 2,377 patients from 7 studies, identified pre-treatment sarcopenia as an independent predictor for worse OS in patients with rectal cancer (HR 2.37; p = 0.02) [31]. Another important aspect is also related to the rate of muscle mass loss during treatment. Regarding that, a recent systematic review confirmed this association between sarcopenia and OS, also introducing the concept that a rapid change of muscle mass during RT could lead to a worse prognosis [38]. These data confirmed the need for a timely body composition assessment aiming to detect low muscle mass early and thus perform an early nutritional and physical intervention. Sarcopenic obese (SO) people, recently defined by ESPEN/EASO as people affected by sarcopenia and obesity, are growing in number, with a mounting increase in related diseases [13]. In our population, about 10 % of obese patients were SO. At present, the exact mechanism of SO in patients with cancer is unclear, but it is probably due to the higher levels of systemic inflammatory markers and insulin resistance, which affects fat metabolism and increases the loss of muscle mass resulting in SO [39], [40]. A recent paper by *Gaoand* and colleagues pooled 38 studies including more than 10,000 patients, identified a prevalence of sarcopenic obesity in 20 % of oncological patients, associated with worse OS (HR 1.82), recurrence-free survival (HR 2.10), DFS (HR 1.94), and postoperative complications (OR 3.01) [39]. Our study showed univariate analysis a worse outcome in terms of OS in sarcopenic obesity (HR 2.83, 95 % CI 1.24–6.45; p = 0.01). Interestingly, a low VAT was identified as a predictor of worse DFS when a subgroup analysis was performed in men, according to the only other paper in the literature that explored this issue, in which the Authors hypothesized a possible explanation in the testosterone role [28]. Prior research has demonstrated that visceral obesity is linked to reduced testosterone levels in men, whereas women with high testosterone levels exhibit a positive association with visceral fat accumulation [41]. Since elevated levels of testosterone have been correlated with an increased risk of death after cancer in both men and women [42], this could lead us to hypothesize a divergent effect of visceral adipose tissue on survival in men and women.

Furthermore, a higher SMI resulted associated with better LC (HR 0.96, 95 % CI 0.93–0.99, p: 0.04). The correlation between tumor control and muscle mass quantity in rectal cancer patients represents a complex interplay with potential clinical implications. On one hand, the augmented muscle mass may contribute to enhanced motor control during therapeutic interventions. On the other, the presence of greater muscle mass is linked to improved blood flow, nutrient delivery, and metabolic activityin the specific context of rectal cancer, where nCRT is the gold. standard for treatment, the enhanced circulation and nutrient supply may create a more conducive microenvironment for effective tumor response, while the heightened metabolic activity associated with increased muscle mass might also contribute to an environment less favorable for tumor progression. Moreover, the role of muscle mass extends beyond its immediate mechanical and metabolic functions. Skeletal muscle is recognized as an endocrine organ capable of producing various cytokines, such as interleukins and insulin-like growth factors, and myokines that can influence tumor behavior and response to treatment, modulating the tumor microenvironment.

To our best knowledge, we presented a work involving the highest number of LARC patients undergoing RT in the literature. On the other hand, our work has some limitations: the retrospective and monocentric nature of the study, which could potentially lead to some misinterpretation of data; the lack of information among pretreatment weight loss, inflammatory markers, and comorbidities; the lack of correlation between nutritional status and postoperative complications or TME quality.

## 5 Conclusions

In summary, this retrospective, observational, study, performed on 628 LARC patients treated with RT, showed that: i) higher BMI and lower SMI impact on RT interruption; ii) higher MD is associated with better OS; iii) higher VAT is associated to worse DFS; iv) higher SMI is associated with better LC.

Future prospective and well-designed studies on the impact of body composition on cancer outcomes are needed. Furthermore, collaboration between radiation oncologists and clinical nutritionists should be implemented, to predict early management of LARC patients with body composition during radiotherapy treatment.

## CRediT authorship contribution statement

**Giuditta Chiloiro:** Conceptualization, Writing – original draft. **Marco Cintoni:** Conceptualization, Writing – original draft. **Marta Palombaro:** Methodology, Writing – original draft. **Angela Romano:** Data curation, Writing – review & editing. **Sara Reina:** Investigation, Writing – review & editing. **Gabriele Pulcini:** Formal analysis. **Barbara Corvari:** Methodology. **Silvia Di Franco:** Formal analysis. **Elisa Meldolesi:** Methodology. **Gabriele Egidi:** Data curation. **Futura Grassi:** Data curation. **Pauline Raoul:** Data curation. **Emanuele Rinninella:** Resources. **Antonio Gasbarrini:** Supervision. **Maria Cristina Mele:** Supervision. **Maria Antonietta Gambacorta:** Supervision.

## Declaration of Competing Interest

The authors declare that they have no known competing financial interests or personal relationships that could have appeared to influence the work reported in this paper.
