# Supplementary material for: Impact of body composition parameters on radiation therapy compliance in locally advanced rectal cancer: A retrospective observational analysis
Source: Clin Transl Radiat Oncol. 2024 Apr 27;47:100789. doi: 10.1016/j.ctro.2024.100789 (PMC11089307; doi:10.1016/j.ctro.2024.100789)
Supplement: Supplementary Data 1 [file mmc1.docx]

**Figure S1.** Correlation between Body Mass Index (BMI) and Skeletal Muscle Index (SMI)

**Figure S2. Kaplan-Meier Curve for Overall Survival (OS) for the entire population**

**Figure S3. Kaplan-Meier Curve for Disease-Free Survival (DFS) for the entire population**

**Figure S4. Kaplan-Meier Curve for Local Control (LC) for the entire population**

**Figure S5. Overall Survival for patients affected with (red) or without (blue) sarcopenic obesity**

***HR: 2.83 [95% CI (1.24-6.45)]; p: 0.01***

**Figure S6. Overall Survival for patients with higher MD (≥ 28.6; red) or lower (<28.6; blue)**

***HR: 0.63 [95% CI (0.44-0.91)]; p: 0.001***

**Figure S7. Overall Survival for sarcopenic patients by Body Mass Index Classes**

**Figure S8. Disease-Free Survival for patients with higher MD (≥ 144.35; red) or lower (<144.35; blue)**

***HR: 1.65 [95% CI (1.06-2.61)]; p: 0.0029***

**Data only for the male population*

**Figure S9. Local Control Survival for patients with higher SMI (≥ 44.05; red) or lower (<44.05; blue)**

***HR: 0.96 [95% CI (0.93-0.99)]; p: 0.02***
