# Supplementary material for: Impact of body composition parameters on radiation therapy compliance in locally advanced rectal cancer: A retrospective observational analysis
Source: Clin Transl Radiat Oncol. 2024 Apr 27;47:100789. doi: 10.1016/j.ctro.2024.100789 (PMC11089307; doi:10.1016/j.ctro.2024.100789)
Supplement: Supplementary Data 2 [file mmc2.docx]

**Table S1. DFS**

|  | HR | p | HR | p |
| --- | --- | --- | --- | --- |
| Age | ***1.02 (1.01-1.04)*** | ***0.036*** | ***1.01 (1.00-1.03)*** | ***0.03*** |
| Female | 0.93 (0.69-1.27) | 0.69 |  |  |
| Weight | 0.99 (0.98-1.01) | 0.62 |  |  |
| Height | 1.00 (0.94-1.05) | 0.90 |  |  |
| BMI | 0.99 (0.95-1.02) | 0.61 |  |  |
| Obesity | 1.10 (0.79-1.54) | 0.56 |  |  |
| SMA | 0.99 (0.98-1.03) | 0.56 |  |  |
| SMI | 0.99 (0.97-1.01) | 0.27 |  |  |
| Low SMI* | 0.74 (0.51-1.07) | 0.11 |  |  |
| MD | 0.99 (0.97-1.02) | 0.38 |  |  |
| Low MD^#^ | 0.88 (0.65-1.19) | 0.41 |  |  |
| IMAT | 0.98 (0.95-1.03) | 0.59 |  |  |
| VAT | ***1.02 (1.01-1.03)*** | ***0.04*** | ***1.02 (1.01-1.03)*** | ***0.021*** |
| Low VAT° | 1.11 (0.82-1.51) | 0.49 |  |  |
| SAT | 0.99 (0.98-1.01) | 0.36 |  |  |
| Sarcopenic Obesity | 1.85 (0.86-3.95) | 0.11 |  |  |
| cT |  |  |  |  |
| T2 | Ref | |  |  |
| T3 | 0.94 (0.52-1.65) | 0.83 |  |  |
| T4 | 1.37 (0.77-2.45) | 0.27 |  |  |
| N+ | 1.02 (0.64-1.63) | 0.92 |  |  |
| N extramesorectal | 1.08 (0.77-1.50) | 0.63 |  |  |
| MRF | ***1.43 (1.06-1.94)*** | ***0.017*** | ***1.63 (1.15-2.29)*** | ***0.005*** |
| pCR | ***0.50 (0.33-0.76)*** | ***0.001*** | ***0.15 (0.03-0.64)*** | ***0.01*** |
| TRG≥3 | 1.22 (0.87-1.71) | 0.23 |  |  |
| RT interruption | 1.10 (0.77-1.56) | 1.58 |  |  |
| Surgical Resection  (R1/2 versus R0) | 1.18 (0.48-2.88) | 0.71 |  |  |

*Abbreviations*

SD: standard deviation; BMI: Body mass index; SMA: Skeletal muscle area; SMI: Skeletal muscle index; MD: Muscle Density; HU: Hounsfield Unit; IMAT: Intramuscular adipose tissue; VAT: visceral adipose tissue; SAT: subcutaneous adipose tissue.

**: SMI<52.4 cm^2^/m^2^ in men and SMI<38.5 cm^2^/m^2^ in women*

*^#^: MD<28.6 HU*

*°: VAT<160 cm^2^ for men and VAT<80 cm^2^ for women*

**Table S2. Local Control**

|  | HR | p | HR | p |
| --- | --- | --- | --- | --- |
| Age | ***1.03 (1.01-1.06)*** | ***0.02*** | ***1.03 (1.01-1.06)*** | ***0.03*** |
| Female | 1.12 (0.65-1.89) | 0.68 |  |  |
| Weight | 0.99 (0.97-1.02) | 0.86 |  |  |
| Height | 0.99 (0.96-1.03) | 0.95 |  |  |
| BMI | 1.09 (0.78-1.51) | 0.60 |  |  |
| Obesity | 0.88 (0.47-1.64) | 0.69 |  |  |
| SMA | 0.99 (0.98-1.00) | 0.11 |  |  |
| SMI | ***0.96 (0.93-0.99)*** | ***0.02*** | ***0.96 (0.93-0.99)*** | ***0.04*** |
| Low SMI* | 0.96 (0.53-1.75) | 0.92 |  |  |
| MD | 0.98 (0.94-1.01) | 0.23 |  |  |
| Low MD^#^ | 0.87 (0.51-1.48) | 0.60 |  |  |
| IMAT | 0.99 (0.98-1.00) | 0.45 |  |  |
| VAT | 0.99 (0.98-1.01) | 0.93 |  |  |
| Low VAT° | 1.22 (0.72-2.07) | 0.46 |  |  |
| SAT | 0.98 (0.97-1.01) | 0.56 |  |  |
| Sarcopenic Obesity | 1.39 (0.33-5.69) | 0.65 |  |  |
| cT |  |  |  |  |
| T2 | Ref | |  |  |
| T3 | 0.45 (0.21-1.09) | 0.07 |  |  |
| T4 | 0.67 (0.29-1.52) | 0.34 |  |  |
| N+ | ***0.45 (0.24-0.86)*** | ***0.01*** | 0.74 (0.31-1.78) | 0.51 |
| N extrameso | 1.62 (0.93-2.81) | 0.08 |  |  |
| MRF | 0.83 (0.48-1.45) | 0.52 |  |  |
| pCR | ***0.36 (0.15-0.85)*** | ***0.02*** | ***0.38 (0.16-0.91)*** | ***0.03*** |
| TRG≥3 | 1.58 (0.84-2.95) | 0.16 |  |  |
| RT Interruption | 1.31 (0.72-2.36) | 0.36 |  |  |
| Surgical Resection  (R0/R1 *vs* R2) | 2.57 (0.79-8.30) | 0.11 |  |  |

*Abbreviations*

SD: standard deviation; BMI: Body mass index; SMA: Skeletal muscle area; SMI: Skeletal muscle index; MD: Muscle Density; HU: Hounsfield Unit; IMAT: Intramuscular adipose tissue; VAT: visceral adipose tissue; SAT: subcutaneous adipose tissue.

**: SMI<52.4 cm^2^/m^2^ in men and SMI<38.5 cm^2^/m^2^ in women*

*^#^: MD<28.6 HU*

*°: VAT<160 cm^2^ for men and VAT<80 cm^2^ for women*
